# How does the quality of life and the underlying biochemical indicators correlate with the performance in academic examinations in a group of medical students of Sri Lanka?

**DOI:** 10.3402/meo.v19.22772

**Published:** 2014-02-20

**Authors:** Manjula Hettiarachchi, Chathuranga Lakmal Fonseka, Priyanka Gunasekara, Prasanjanie Jayasinghe, Dasun Maduranga

**Affiliations:** 1 Faculty of Medicine, University of Ruhuna, Galle, Sri Lanka; 2 Coronary Care Unit, Teaching Hospital, Karapitiya, Galle, Sri Lanka

**Keywords:** exam stress, quality of life, cortisol, thyroxine, Sri Lanka

## Abstract

**Background:**

Individual variation of examination performance depends on many modifiable and non-modifiable factors, including pre-examination anxiety. Medical students’ quality of life (QoL) and certain biochemical changes occurring while they are preparing for examinations has not been explored.

**Purpose:**

We hypothesize that these parameters would determine the examination performance among medical students.

**Methods:**

Fourth-year medical students (*n*=78) from the University of Ruhuna, Sri Lanka, were invited. Their pre- and post-exam status of QoL, using the World Health Organization Quality of Life (WHOQOL-BREF) questionnaire, and the level of biochemical marker levels (i.e., serum levels of thyroid profile including thyroglobulin, cortisol and ferritin) were assessed. Differences between the scores of QoL and serum parameters were compared with their performance at the examination.

**Results:**

The mean QoL score was significantly lower at pre-exam (56.19±8.1) when compared with post-exam (61.7±7.1) levels (*p*<0.001). The median serum TSH level prior to the exam (0.9 mIU/L; interquartile range 0.74–1.4 mIU/L) was significantly lower (*p*=0.001) when compared with the level after the exam (median of 2.7 mIU/L; IQR 1.90–3.60). The mean±SD fT4 level was significantly higher before the exam (19.48±0.4 pmol/L at study entry vs. 17.43±0.3 pmol/L after the exam; *p*<0.001). Median serum ferritin (SF) level prior to the exam (43.15 (23.5–63.3) µg/L) was significantly lower (*p*≤0.001) when compared with after-exam status (72.36 (49.9–94.9) µg/L). However, there was no difference in mean serum cortisol levels (16.51±0.7 at pre-exam and 15.88±0.7 at post-exam, respectively; *p*=0.41).

**Conclusions:**

Students had higher fT4 and low ferritin levels on pre-exam biochemical assessment. It was evident that students who perform better at the examination had significantly higher QoL scores at each domain tested through the questionnaire (Physical health, Psychological, Social interaction and Environment). The higher the QoL scores, the better the grades were. It was also found that students who failed exhibited profound differences in the QoL score.

## Background

Improving examination performances of students and reducing their failures are among the key expectations of teachers and students in any educational system. Finding out contributing factors for student's exam performance is important so that remedial measures can be taken to achieve desired results (1). Stress (2) and burnout (3) are noteworthy factors affecting drop-out rates caused by heavy academic load, stress of frequent exams (4) and lack of relaxation. Better understanding of the quality of life (QoL) issues could assist the students to manage their stress in order to perform better in their exams. Further, the abbreviated version of the World Health Organization Quality of Life (WHOQOL-BREF) has recently proved to be helpful to assess health related QoL issues among medical students (5).

The WHOQOL-BREF is an international cross-culturally comparable QoL assessment instrument, which is available in different languages for both developed and developing countries (5). The questionnaire includes four domains: physical health, psychological health, social relations, and environment. The scores are transformed into a linear scale between 0 and 100, with 0 being the least favorable and 100 being the most favorable. The Field Centre for WHOQOL, University of Ruhuna, Sri Lanka, has already validated the translated version (local language is Sinhala) of WHOQOL-BREF (6).

Psychological stress has short-term as well as long-term effects. In the short term, it increases corticotrophin releasing hormone secretion and the basal cortisol level so that the body acclimatizes itself to a hypercatabolic state. This also increases the metabolic demands of the body and may causes changes in the hypothalamo-pituitary axis, thus making changes in hormone levels including the thyroid status (7). Cortisol and thyroxine are two vital hormones of the human body and studies have found an association of stress with the diseases of the thyroid (8). Thyroglobulin (Tg) level can be checked in these individuals to detect the severity of damage to the thyroid gland (9). It has been found that clinical and subclinical hypothyroidism as well as hyperthyroidism in the middle-aged are both associated with decreased cognitive function, especially memory, attention, and reaction time (10). Iron deficiency has been found to be associated with poor cognitive development and supplementation has shown to normalize cognitive functions in certain age groups of people (11). Serum ferritin (SF) level is a powerful and freely available test to detect iron deficiency (12), and it detects the depletion of iron stores early since actual drops in hemoglobin concentration occur later (13).

Most students had experienced either academic and/or psychosocial stressors leading to deteriorating the QoL. Among academic stressors, ‘tests/exams’ were the chief sources of stress. Despite this, test/exams are important in the academic training as a standard for evaluation/assessment. The purpose of the present study is to assess relationship of QoL and biochemical stress markers (i.e., serum levels of thyroid profile, cortisol, and ferritin) on the examination performance among fourth-year medical students. This data will be useful in the management of stress and to make behavioral changes to improve their exam performance.

## Methods

The fourth-year medical undergraduates (*n*=129) at the University of Ruhuna were invited to participate in this prospective study, consisting of 60 (48.5%) females with the remainder male. They had already completed two examinations (2nd MBBS and 3rd MBBS part 1) in the preceding 3 years, followed by two further years of clinical training. At the time of this study, they were preparing for their fourth-year examination, namely 3rd MBBS part 11 examination. Passing of this exam is mandatory to be eligible for the final examination in clinical subjects (Medicine, Surgery, Pediatrics, Gynecology & Obstetrics, and Psychiatry) in the fifth year. We made the announcement of this study 3 months prior to the examination, and the tentative schedule of data collection was also displayed. A total of 78 students participated (36 females) and completed the study after obtaining informed written consent. The study received approval from the Ethical Review Committee of the Faculty of Medicine, University of Ruhuna, and was conducted in 2011.

### Selection criteria

- Medical students sitting their 3rd MBBS examination for the first time.

### Exclusion criteria

- Students who are already on treatment for thyroid disorders.
- Students experiencing other major stressful events.
- Students with acute febrile illnesses and any other illnesses.

The baseline level of QoL was collected by using a Sinhala (local language) standardized version of the WHOQOL-BREF (6) 2–3 weeks prior to the main examination, while they were on study leave. We chose this time period expecting some observable examination stress by that time and to ensure our interventions would not cause further anxiety to the detriment of their exams if conducted too close to the dates of exams. This questionnaire has 26 items on a five-point Likert scale, which includes: two global items about QoL and health, respectively, and 24 items relating to four domains calculated as the sum of seven items for physical, six for psychological, three for social and eight for environmental QoL. Further, 5.0 mL of venous blood sample was obtained from each subject to assess serum levels of random cortisol (Cor), free thyroxine (free T_4_), thyroid-stimulating hormone (TSH), Tg, and SF levels. Once collected, serum was separated and stored in the Radioimmunoassay laboratory of the Nuclear Medicine Unit at −80°C until analysis. The questionnaire and serum samples were given an enrollment number to each participant. The same procedure was carried out 3–4 weeks after the examination and before the examination results were given to minimize the bias created by the actual results. The enrollment number was used to identify matched pairs of pre- and post-exam WHOQOL-BREF and serum samples. Further, once the examination results were published, students were requested to mention their individual results (referred, pass, or pass with a class) against the enrollment number. A total score of >50 was determined to be a ‘pass’, and below that was considered ‘referred’. When the total score fell between 50 and 60, it was a ‘simple pass’; whereas to be considered a ‘pass with a class’, the student should obtain marks >60. The curriculum they followed was the traditional medical curriculum.

## Statistical analysis

Where appropriate, data were expressed as means and standard deviations. In one sample, a Kolmogorov–Smirnov test was used to investigate whether the concentrations of serum indicators were normally distributed. The distributions of TSH, Tg, and SF concentrations were skewed, thus they were log transformed in all calculations. For presentation, these variables were transformed back to the original scale and presented as median and interquartile ranges (IQR). Paired *t*-tests were used to study within-group treatment effects. Between-group treatment effects (mean change in scores in pre- vs. post-exam) between level of scores on four domains of QoL and serum parameters were compared using repeated measures design. Pre-exam values of respective parameters were also included in the analysis as a covariant for between-subject factors to correct for their possible confounding influence on the change. QoL domains were further analyzed among proportions of subgroups (exam failures vs. better performers). Probability values <0.05 were considered as statistically significant. All data analyses were conducted using SPSS v.15.0 (SPSS^®^ 15.0, Chicago, IL, USA). SF was used as a marker of acute phase reaction to exclude acute/chronic infection (SF>400 µg/L).

## Results

A total of 78 medical students out of 129 of the batch participated and completed the study. There were no dropouts at the post-examination data collection. Table 1 summarizes the basic characteristics of the study sample at 2–3 weeks prior to the main exam (pre-exam) and 3–4 weeks after the examination (post-exam). During this period, other than serum cortisol and Tg levels, all investigated parameters showed a significant difference when compared with the baseline value of each indicator. The median serum TSH level prior to the exam (0.9 mIU/L; IQR 0.74–1.4 mIU/L) was significantly (*p*=0.001) lower when compared with after-exam status (median of 2.7 mIU/L; IQR 1.90–3.60). The fT4 level prior to the exam (19.48±0.4 pmol/L) was higher when compared with the fT4 level after the exam (17.43±0.3 pmol/L; *p*<0.001; Table 1). The baseline Tg level had significant correlations at baseline with TSH (*r*=0.34, *p*=0.002), fT4 (*r*=−0.26, *p*=0.02) and SF (*r*=−0.25, *p*=0.03). There were no significant differences in TSH and fT4 levels between males and females (data not shown).

**Table 1:** Basic characteristics of the study sample^1^

Parameter	Before exam	After exam	*p*^a^
QoL score	56.19 (8.1)	61.7 (7.1)	<0.001
Serum TSH	0.9 (0.7, 1.4)	2.7 (1.9, 3.6)	<0.001
Serum free T_4_	19.48 (0.4)	17.43 (0.3)	<0.001
Serum cortisol	16.51 (0.7)	15.88 (0.7)	0.41
Serum ferritin	43.15 (23.5, 63.3)	72.36 (49.9, 94.9)	<0.001
Serum thyroglobulin	0.15 (0.08, 0.5)	0.15 (0.06, 0.5)	0.69

1 78 students participated in this study. Results presented as mean (SD) or median (interquartile range).

a Derived from paired sample *t*-test.

It was worthy to note that median SF level prior to the exam (43.15 µg/L) was significantly lower (*p*≤0.001) when compared to the level after the exam (72.36 µg /L). Female students had baseline mean±SEM SF (21.70±2.55 µg/L) which was increased by two-fold (45.73±4.27 µg/L; *p*<0.001) after the main exam. Male students had a baseline level of 62.44±4.16 µg/L and it was increased to 100.56±7.29 µg/L (*p*<0.001).

The correlation coefficients of serum biochemical parameters on QoL level (before and after examination) are presented in Table 2. Both serum TSH (*r*=−0.194; *p*=0.04) and cortisol (*r*=−0.012; *p*=0.46) had negative correlations before examination. Further, SF (*r*= 0.212; *p*=0.03) had a significant correlation with baseline QoL. However, after the examination only serum TSH (*r*=−0.190; *p*=0.05) had a marginally significant negative correlation with QoL. Further, other parameters such as serum cortisol (*r*=−0.08; *p*=0.24), SF (*r*=−0.04; *p*=0.35) also showed that a higher QoL score was associated with lower serum biochemical status.

**Table 2:** Correlations of biochemical parameters on QoL score during examinations^1^

Parameter	Before exam QoL	After exam QoL
Serum TSH	−0.194 (0.04)	−0.190 (0.05)
Serum free T_4_	0.068 (0.26)	0.010 (0.47)
Serum ferritin	0.212 (0.03)	−0.035 (0.38)
Serum cortisol	−0.012 (0.46)	−0.081 (0.25)
Serum thyroglobulin	0.037 (0.37)	0.182 (0.06)

1 Results presented as correlation coefficient (one-tailed *p* value).

The exam results were compared with main four domains of the QoL scores and are presented in Table 3. A total of 54 students were successful in achieving the goal of completing the examination in their first attempt. The WHOQOL-BREF has four main domains (Physical health, Psychological, Social interaction and Environment) to be analyzed and the maximum score that can be obtained in each domain is 20. The ‘pass’ students had significantly higher scores in each domain when compared with their counterparts, who were ‘referred’ at the examination (*n*=24; Table 3). When the WHOQOL-BREF was reintroduced after the examination, both groups showed higher scores in each domain with no difference between them except for the total score of the referred group, who exhibited significant improvement (Table 3). The biochemical analysis revealed that there was no difference among them when compared with their exam performances (Table 4).

**Table 3:** Exam performance and the analysis of GHQ in different domains among medical students^1^

Parameter	Referred (*n*=24)		Pass (*n*=54)
Domain 1: Physical health	Pre	12.6 (2.1)	13.9 (2.2)^a^
	Post	14.7 (2.4)	15.2 (1.8)
Domain 2: Psychological	Pre	13.2 (2.1)	14.3 (2.0)^a^
	Post	14.5 (2.1)	15.5 (1.7)
Domain 3: Social relationship	Pre	13.5 (2.6)	14.7 (2.6)
	Post	15.5 (3.4)	16.0 (2.0)
Domain 4: Environment	Pre	13.6 (2.1)	14.8 (2.2)^a^
	Post	15.3 (2.1)	15.8 (1.7)
Total score	Pre	52.9 (7.8)	57.8 (7.8)^a^
	Post	60.1 (8.9)^b^	62.5 (6.1)

1 Results presented as mean (SD).

a There were significant differences at baseline (*p*=0.05) between groups.

b Mean change in total score improved significantly (repeated measures, *p*=0.05).

**Table 4:** Exam performance and the biochemical analysis among medical students^1^

Parameter	Referred (*n*=24)		Pass (*n*=54)
Serum TSH	Pre	1.20 (0.85, 1.70)	0.90 (0.60, 1.40)
	Post	2.65 (2.4, 4.4)	2.75 (1.9, 3.5)
Serum free T_4_	Pre	18.76 (2.3)	19.80 (3.7)
	Post	17.48 (2.0)	17.40 (2.6)
Serum ferritin	Pre	35.29 (18.4, 53.7)	43.97 (27.5, 70.1)
	Post	67.28 (44.7, 91.1)	73.96 (50.5, 95.2)
Serum cortisol	Pre	16.56 (6.2)	16.48 (5.6)
	Post	16.58 (5.4)	15.57 (6.9)
Serum thyroglobulin	Pre	0.16 (0.1, 0.9)	0.14 (0.08, 0.5)
	Post	0.21 (0.1, 1.7)	0.12 (0.05, 0.35)

1 Results presented as mean (SD) or median (interquartile range).

Out of 54 students who got through the main exam, nearly 50% of them (*n*=24) had very good passes (i.e., classes with distinctions). Therefore, the WHOQOL-BREF scores were re-analyzed between two groups of students (simple pass vs. pass with a class). It was evident that students who perform better at the examination had significantly higher QoL scores at each domains of study (data were not shown). Further, those who got a simple pass had significant improvement (*p*<0.05) in the total score at the post-exam assessment (61.4±7.0) when compared with their pre-exam QoL score (54.7±7.2).

## Discussion

This would be a novel study on psychosocial health and study related biochemical analysis among medical students who were about to complete their degree program. From this study, we were able to demonstrate that before an academic examination, a relative hyperthyroid status existed among medical students. This may have occurred as an adaptation mechanism to face exams as a result of a fight or flight response. However, biochemically no significant increment of cortisol was seen in our study, which may be due to the fact that our sample collection was too early to rise (2–3 weeks prior to the examination). In a previous study where serum cortisol and ACTH levels were measured in two groups (normal and anxious based on anxiety standard questionnaire), both groups had significant increased levels, when analyzed 20 min before the examination (14). Although previous studies (15, 16) have attributed the nervousness and related symptoms to the cortisol levels, this study shows that those symptoms may occur due to the increased metabolic status of the body created by increased thyroid hormone levels.

The current study evaluated the relation between SF level and QoL during an exam stress period. It has been shown that mean ferritin levels were towards the lower limit of the normal range in pre-exam analysis. However, it was almost doubled after the examination. So, the relative depletion of iron stores might also contribute to depressive or anxiety symptoms experienced in the pre-exam state. A clinical trial conducted in Sweden showed that iron supplementation could diminish depressive symptoms in students compared to the control group (17). In another study in the US, the prescription of iron led to decreased depression in mothers with iron deficiency anemia compared to the controls (18). The fact that the mean SF level was lower in exam ‘referred’ students than in ‘pass’ ones further strengthens the belief in the possible role of iron in brain function and the establishment of a depressive mood. Iron plays an important role in the oxygenation of brain parenchyma and the synthesis of many neurotransmitters and enzymes of the nervous system.

The amount and severity of stress experienced by medical students may vary according to the settings of the medical school, the curricula, examination systems, etc. Previous studies from medical schools in different countries have reported that academics/exams are common sources of stress among medical students (19–22). These studies have used different methods to measure stress. This limits the comparability among these studies. Therefore, we chose the WHOQOL-BREF since this instrument has been documented for its reliability and validity. All of these tools have demonstrated that students had a global response to a wide range of potential stressors rather than being limited to a few specific items.

In a recent study, the psychometric properties of the WHOQOL-BREF when applied to medical students (5), demonstrated adequate internal consistency. They have detected a strong ceiling effect on three items (pain and discomfort; dependence on medicinal substances and medical aids; and mobility) in the physical health domain and it appeared to be a common occurrence when collecting data from samples of younger people (23). We were unable to verify this finding as our focus is on QoL related to examination stress.

Stowell (24) referred to a commonly accepted fact that the stress level in students is increased during academic examinations and the label ‘academic examination stress’ covers a wide range of situations that may have very different psychological and immunological consequences. Exam anxiety is the emotional reaction that some students face before exams. The fear is not irrational, but excessive fear interferes with performance. Many researchers suggest that a little worry is good for students because it keeps them task oriented; however, excessive worry can be very debilitating and can interfere with their results if not managed for students (22–28) and to redesign leisure activities during this period. All four domains of study in WHOQOL-BREF were identified as important sources of stress prior to the exam. This may be due to time constraints for self, family, friends, and entertainment due to the demanding medical curriculum. A study from the United States has recommended that teaching stress management and self-care skills for medical students may prove to be beneficial (29). There is a need to look at the applicability of such measures, which are feasible in our medical school setting.

## Limitations

This study has a number of limitations including generalizability. Nearly 40% of students (*n*=51) in this batch did not respond to our request to participate, which possibly contributed to responder bias, as students who were more stressed due to the pressure of exams did not respond. Alternatively, students who were least stressed may have decided that they have little to contribute and so did not respond. Potential confounding variables such as gender, social status, sleep, diet, effectiveness of preparation for examinations were not considered in the data analysis. However, almost all students were given the hostel facilities within the university premises starting so that most of these variables (other than gender) were likely to be even among study subjects. Though participation was anonymous, students have to divulge their exam results. This may be a sensitive and personal aspect of the study. Future studies should use larger sample size, qualitative measurement of anxiety levels and comparison of groups of students with higher and lower anxiety levels for contributory factors to validate the results.

## Conclusions

There is a considerable amount of deterioration of general health and QoL, which produces changes in biochemical parameters among medical students due to exams. It was also reflected that QoL was more affected in exam failures. The QoL level may reflect not only the academic performances but also some aspects of student health. The individual students should be identified and helped, as stress in examinations cannot be eliminated.

## Author contributions

MH has made contributions to concept/design, acquisition of data, analysis and interpretation of data; was involved in drafting the manuscript/revising it critically for important intellectual content; and has given final approval of this version to be published.

LF has made contributions to concept/design, acquisition of data, analysis and interpretation of data; was involved in drafting the manuscript/revising it critically for important intellectual content; and has given final approval of this version to be published.

PG has made substantial contributions to concept/design, analysis and interpretation of data; was involved in drafting the manuscript/revising it critically for important intellectual content; and has given final approval of this version to be published.

PJ has made substantial contributions to concept/design, acquisition of data; involved in drafting the manuscript/revising it critically for important intellectual content; and has given final approval of this version to be published.

DM has made substantial contributions to concept/design, acquisition of data; was involved in drafting the manuscript/revising it critically for important intellectual content; and has given final approval of this version to be published.
